# Current Status of Protein Biomarkers in Urolithiasis—A Review of the Recent Literature

**DOI:** 10.3390/jcm12227135

**Published:** 2023-11-16

**Authors:** Aleksandra Lasota, Anna Wasilewska, Agnieszka Rybi-Szumińska

**Affiliations:** Department of Pediatrics and Nephrology, Medical University of Bialystok, Waszyngtona 17, 15-297 Bialystok, Poland; anna.wasilewska@umb.edu.pl (A.W.); arybiszuminska@gmail.com (A.R.-S.)

**Keywords:** urolithiasis, children, protein biomarkers

## Abstract

Urolithiasis is an increasingly common clinical problem worldwide. The formation of stones is a combination of metabolic status, environmental factors, family history and many other aspects. It is important to find new ways to quickly detect and assess urolithiasis because it causes sudden, severe pain and often comes back. One way to do this is by exploring new biomarkers. Current advances in proteomic studies provide a great opportunity for breakthroughs in this field. This study focuses on protein biomarkers and their connection to kidney damage and inflammation during urolithiasis.

## 1. Introduction

Urolithiasis (i.e., kidney stone disease) remains a global public health problem with increasing prevalence [1,2]. This disease is spreading in developed countries and affecting both adults and children, becoming a problem for society.

Women have a higher risk of early onset lithiasis, with a tendency to have recurrences. Late stone formation occurs more frequently in those with a BMI > 30 [3].

Urolithiasis is a disease in which deposits are formed in different parts of the urinary tract: the pyelocalyceal system, the ureter or even the bladder. The pathogenesis of the disease is complex and multifactorial. An imbalance between promoter (like calcium, oxalic acid, uric acid, cystine, cell fragments) and inhibitor (like magnesium, citrates, zinc, glycosaminoglycans, uromodulin, osteopontin, osteocalcin, bicunin) concentrations, along with specific physicochemical conditions, increases the risk of lithiasis. A very important aspect is the pH of the urine, which, depending on the type of deposits, can determine their precipitation. In the case of uric acid stones, an acidic environment stimulates their precipitation. The formation of stones is also influenced by antibiotic therapy and the reduction in intestinal colonization with the bacterium Oxalobacter formigenes. Indeed, this organism uses oxalic acid as an energy source, reducing its absorption in the gastrointestinal tract [4]. Also, a sedentary lifestyle, a diet high in animal-based protein with a high intake of salt and a low intake of water and the presence of obesity can increase the risk of stone crystallization. The chance of urolithiasis is 2–16 times higher in patients with a family history of stone disease [5]. We categorize urolithiasis into different types based on deposit composition and metabolic abnormalities. The most common type, accounting for 70–80% of cases, is calcium oxalate (CaOx). Uric acid urolithiasis is caused by excessive excretion of uric acid as a metabolite of purine metabolism. Cystine is associated with cystinuria. Magnesium ammonium phosphate (struvite) accompanies urinary tract infection. Other less common types include calcium phosphate, xanthine and 2,8-dihydroxyadenine [6]. On suspicion of urolithiasis, a comprehensive metabolic diagnosis is required to identify risk factors. The most common include hypercalciuria, hypocitraturia, hyperuricosuria and hyperoxaluria [5,7]. Most cases of deposits in the urinary tract are diagnosed during a renal colic episode or incidentally during an abdominal ultrasound performed because of non-specific symptoms, such as abdominal pain. Ultrasonography is the most important imaging study for diagnosing urolithiasis. It is widely available and sensitive and does not involve radiation doses. In the case of diagnostic problems, such as difficult localization or size of the deposit, spiral computed tomography without contrast should be performed [8,9]. Metabolic assessment is important after stone expulsion and should be repeated multiple times. It includes blood tests, urine samples and a 24 h urine collection to test for crystallization promoters and inhibitors. Analysis of the composition of the expelled stone is also an extremely important aspect. We currently have three methods: infrared spectroscopy, polarization microscopy and X-ray diffraction [8,10]. Based on the collected results, mainly from the metabolic assessment, targeted prophylaxis and conservative treatment can be implemented. Up to 80% of the deposits are expelled spontaneously, mainly those with a small diameter of up to 5 mm. The treatment of urolithiasis can be divided into conservative and invasive. Medical expulsion therapy (MET) relies on analgesics (NSAIDs) and agents that facilitate expulsion: alpha-blockers (doxazosin, tamsulosin, alfuzosin), calcium channel blockers (nifedipine) and corticosteroids. When conservative therapy is unsuccessful, procedures that are as minimally invasive as possible should be implemented. These include extracorporeal shock wave lithotripsy (ESWL), lithotripsy during ureteroscopy (URSL), percutaneous nephrolithotripsy (PCNL) and retrograde intrarenal surgery (RIRS). Because of the efficacy and prevalence of the above methods, classical surgical treatment is a rare choice [8,9]. 

Even though there are traditional diagnostic methods available, researchers are searching for new biomarkers to understand the risk of kidney stone formation and possible complications like AKI, CKD or urosepsis. 

The principal goal of this review is to gather evidence from recent literature about protein biomarkers in urolithiasis patients that might be useful for establishing the diagnosis, monitoring the disease activity and prognosing the outcome of the disease.

## 2. Materials and Methods

### Search Protocol 

This review follows the PRISMA 2020 guidelines (Preferred Reporting Items for Systematic Reviews and Meta-Analyses) [11]. We searched PubMed and Web of Science databases. All records between January 2016 and September 2023 were checked by two independent individuals with the strategy using MeSH (Medical Subject Heading) terms and keywords for the description of population and intervention with the help of the Boolean operators ”or” and “and”. We used the combination of “urolithiasis” OR “kidney stones” AND “protein markers” AND “blood” OR “urine”. The inclusion criteria were as follows: (1) human research, (2) clinical diagnosis of urolithiasis, (3) protein marker obtained in blood or urine, with the use of ELISA method, (4) cohort and case-control investigations and (5) articles published in English in peer-reviewed journal. The exclusion criteria were as follows: (1) animal research, (2) no specific diagnosis of urolithiasis, (3) other markers like DNA/RNA molecules, bacteria and metabolome, (4) presence of factors like severe diseases or treatment that might influence the obtained results, (5) no control group, or it was not precisely chosen, (6) studies not in English and (7) full text not available.

**Figure 1:**
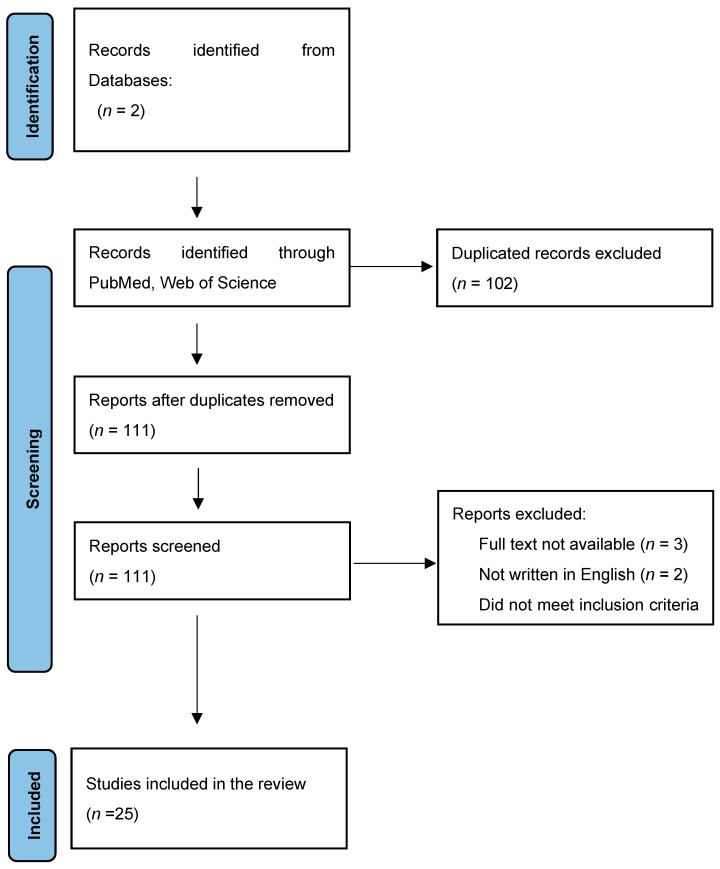
Studies of the last 7 years (2016–2023) included in this review—PRISMA 2020 flow diagram. * Web of Science, PubMed/Medline.

A total of 213 records in the period of the last 7 years (2016–2023) were found, but only 25 were taken into further review. The excluded studies were duplications or did not meet inclusion criteria; for instance, there was no obvious diagnosis of urolithiasis, research was performed on animals, they were “in vitro” studies, and markers were not proteins or obtained in material other than blood or urine. There were also 10 case studies and 9 review studies excluded, 3 were not available as a full-size text, and 2 were not in English (Figure 1).

## 3. Results

The review of protein biomarkers in urolithiasis patients detected a wide range of molecules in their urine and blood. Usually, they were assessed as a panel of a few proteins to get a better insight into their role in the process of stone formation as well as further progression or complications of this disorder. Detailed results of the studies from the years 2016–2023 on protein markers in urolithiasis are organized in Table 1. 

Cystatin C is one of the oldest and best-known proteins that may serve as a kidney function biomarker. It was not a matter of main interest in urolithiasis; however, we found two recent research studies that, among other markers, assessed cystatin C. Kovacevic L. et al. [27] performed interesting proteomic analysis of cystatin C, Neutrophil Gelatinase-associated Lipocalin (NGAL) and lysozyme C in the urine of 24 urolithiasis children and 13 healthy controls. Both cystatin C and NGAL showed significantly higher levels in affected children and nearly significantly in the case of lysozyme C. Hughes S.F. et al. [23] measured cystatin C, NGAL procalcitonin (PCT) and myeloperoxidase (MPO) in the blood of adult patients with urolithiasis to predict the risk of postoperative complications following flexible ureterorenoscopy. The blood samples were taken in time intervals before and after the procedure. Only NGAL showed a significant rise following the operation while no changes in the concentration of cystatin C, PCT or MPO were observed. There are many more recent research studies on NGAL as one of the most promising tubular protein markers in urolithiasis. Hughes S.F. et al. measured [25] NGAL and inflammatory cytokines (interleukins IL-18, IL-10, IL-8, IL-6 and TNF-alfa) in the blood of 12 lithiasis patients after shock wave lithotripsy. The samples were taken before operation and then in time intervals after the procedure. NGAL concentration was significantly increased after the procedure, similar to IL-6 and TNF-alfa. An IL-18 increase was observed but without statistical significance, whereas IL-10 and IL-8 concentrations did not change. Kandur Y. et al. [12] also measured levels of NGAL Kidney Injury Molecule—1 (KIM-1) and IL-18 in the urine of 40 children with nephrolithiasis in comparison to 23 participants with hypercalciuria and 20 healthy peers. Similarly, in this study, uNGAL/cr showed a significant rise in stone formers when compared to controls, while no differences were observed in uKIM-1/cr as well as uIL18/cr. In the study of Tasdemir M. et al. [15], urinary NGAL/cr was evaluated together with urinary KIM-1/cr and N-acetyl-beta-D-glucosaminidase (NAG)/cr in a children cohort with urolithiasis. The measurements were taken three times, in 6-month intervals. Only uNGAL/cr and uNAG/cr showed a significant rise in patients with hydronephrosis, whereas all these markers did not differ between lithiasis participants and healthy controls. In the study of Wang X. et al. [22], we also found the assessment of urinary NGAL as well as Monocyte Chemotactic Protein—1 (MCP-1), 8-isoprostane (8-IP), Liver-fatty acid binding protein (L-FABP), Heart-type fatty acid binding protein (H-FABP), clusterin and osteopontin (OPN) in 30 patients with primary hyperoxaluria and calcium oxalate (CaOx) crystals compared to healthy controls. Higher uNGAL and OPN were positively correlated with eGFR, whereas higher MCP-1 was positively correlated with CaOx supersaturation. In the study of Milisic E. et al. [30], urinary NGAL was a proposed marker of acute kidney injury in lithiasis patients who had extracorporeal shock lithotripsy (ESWL). They measured uNGAL in 62 stone formers before and 6 h, 12 h, 7 days and 3 months post-procedure. The levels were significantly rising with hours post ESWL, achieving the highest values after 12 h. UNGAL was negatively correlated with eGFR. One of the newest reviewed studies from 2023 conducted by Memmos D. et al. [35] on urinary NGAL and other biomarkers of renal injury in lithiasis participants compared the results of standard percutaneous nephrolithotomy (sPCNL) with miniaturized (mPCNL) and retrograde intrarenal surgery (RIRS). It was a randomized clinical trial, including 75 patients. No significant differences were shown in uNGAL/cr and in other markers (uKIM-1/cr or uIL-18/cr) between these subgroups. Within particular subgroups, the highest increases from baseline to 2 h post-procedure were in uNGAL/cr, uKIM-1/cr and uIL-18/cr. No changes in any of these indicators were noticed in patients who had complications, including AKI. Carbohydrate antigen 19-9 (Ca 19-9) was also assessed in patients as a predictor of renal injury due to urolithiasis. Amini E. et al. [13] in 2016 designed a study evaluating CA 19-9 in the serum and urine of 38 stone formers with hydronephrosis who underwent transurethral lithotripsy (TUL) and 24 healthy peers. The median concentration of urinary CA19-9 and serum CA19-9 was 34.0 and 15.0 kU/L in the urolithiasis group and 16.1 and 5.3 kU/L in the healthy peers, respectively (*p* < 0.001). Following successful TUL and hydronephrosis resolution, a significant decline was detected in serum and urinary CA19-9. The duration of ureteral obstruction was associated with higher serum and urinary CA19-9 concentrations, suggesting its predictive value for renal damage associated with urinary tract obstruction. The last reviewed study on kidney function indicators in urolithiasis was performed in China by Xiaohong F. et al. [32] on a very large population of stone formers. They included 10,281 participants divided into subgroups according to the results of a kidney ultrasound: 1—507 patients who had unilateral renal stones; 2—75 patients with bilateral renal stones; and 3—peers without urolithiasis. The participants had detailed metabolic assessments including biochemistry, eGFR (CKD was defined as eGFR < 60 mL/min/1.73 m^2^), albuminuria, glucose, blood pressure as well as urinary NAG/cr and urinary alfa1-MG/cr. It was shown that bilateral stone formers had the biggest proportion of CKD as well as other metabolic disturbances including hypertension and hyperglycemia. They also had higher uNAG/cr and higher alfa1-MG/cr levels in comparison to healthy peers.

Several recent studies assessed inflammatory markers in patients with urolithiasis. Venkatesan S. et al. [14] conducted cross-sectional research on 41 stone formers compared to 41 matched healthy controls assessing serum IL-6 and high-sensitivity C-reactive protein (hsCRP) together with intact parathyroid hormone (iPTH), vitamin D and 24 h urinary calcium and phosphorus excretion. Significant differences considering all evaluated parameters were observed between cases and controls. In the research of Kusumi K. et al. [21], who assessed a profile of 30 urinary cytokines in lithiasis adolescents and healthy peers, IL-13 and Macrophage Inflammatory Protein 1beta (MIP1β) were significantly increased in stone formers, whereas IL-17A was higher in controls. Cilesiz N.C. et al. [26] tried to predict spontaneous ureteral stone passage based on the concentration of procalcitonin. The patients that were able to eliminate the stone spontaneously had significantly lower levels of PCT than those who still had the stone after a 4-week follow-up. With the use of ROC curve analysis, they identified an optimal cut-off PCT value of 160 pg/mL (86.7% sensitivity, 70.8% specificity, *p* < 0.001; AUC 0.788 95% CI 0.658–0.917). Another research study by Taiguo Q. et al. [28] assessed the diagnostic and predictive role of IL-6 and PCT for postoperative urosepsis in 90 lithiasis patients after percutaneous nephrolithotomy. Participants with sepsis had higher IL-6 after 2 h post-surgery and higher IL-6, PCT on postoperative day one and PCT on postoperative day three when compared to patients without septic complications. IL6 was concluded to be a possible predictive marker of early diagnosis for postoperative sepsis. Ramasamy V. et al. [29] evaluated the role of the main inflammatory indicator, CRP, in predicting the outcome of MET for distal ureteric lithiasis. CRP values were higher in stone non-passers when compared to those who eliminated the calculus. Multivariate analysis showed an association between higher CRP > 1.35 mg/dL and the size of the stone >7 mm with MET failure. Wymer K. M. et al. [31] retrospectively reviewed 98 patients with urolithiasis who underwent upper urinary tract decompression to predict the risk of UTI based, within other parameters, on the serum CRP and PCT concentration. It was shown that CRP serum level >21.95 mg/dL and PCT > 0.36 µg/L had a significant predictive role. The newest study of Savin Z. et al. [36] assessed the role of white blood cells (WBC) in morphology, CRP and creatinine serum levels in renal colic patients as predictors of urinary tract obstruction because of the stone. Creatinine 0.95 mg/dL and WBC 10,000 were the most accurate thresholds for predicting the obstruction of the urinary tract.

In the group of protein markers evaluated in urolithiasis are also those that take part in the stone formation. A few of the best known are osteopontin (OPN), bikunin, uromodulin and nephrocalcin. Icer M. et al. [16] conducted a study on 88 participants (44 stone formers and 44 healthy peers) to assess urinary OPN in relation to anthropometric measurements and nutrition intake. OPN levels were lower in affected patients than in controls. Other evaluated parameters also correlated with OPN; however, it was gender-dependent. Urinary OPN excretion was decreased in patients with lithiasis in the study of Jung K. et al. [17] in contrast to increased bikunin in stone formers. These authors did not show a statistically significant increase in urinary calgranulin in affected participants. OPN and other proteins involved in the process of stone formation were subjects of interest in the research of Kovacevic L. et al. [34]. It was a proteomic analysis of several inhibitory protein profiles in children with urolithiasis. The authors confirmed that 17 proteins were significantly decreased in affected patients when compared to controls. Also, five of them (two actins, annexin A5, keratin 6B and serpin B4) were absent in the urine of stone formers. OPN urinary excretion was significantly lower in urolithiasis with hypercalciuria when compared to controls and positively correlated with urinary citrate excretion. The study of Noonin C. et al. [33], who assessed Tamm–Horsfall protein (THP) and its role in calcium oxalate stone formation, revealed that THP concentration-dependently reduced CaOx monohydrate crystal size and inhibited these crystals’ growth, aggregation and further cell adhesion. THP seemed to bind only calcium ions, not oxalate. 

## 4. Discussion

### 4.1. Biomarkers of Tubular Injury

There is a strong link between urolithiasis and renal tubular injury. On the one hand, there is evidence that oxalate and CaOx crystals injure tubular epithelium. 

Research using animals found that having CaOx crystals in the kidney can cause tubular cell damage and result in enzymes and debris in urine [37,38,39].

On the other hand, there are studies suggesting that tubular cell injury may stimulate crystallization. The report of Wiessner J. H. et al. revealed that both individual cells and total tubular monolayer injury exposed cell surfaces resulting in increased affinity for crystal adhesion and their further retention in the collecting duct [40].

Bigger stones, during passage down the ureter, may obstruct renal outflow, causing acute or chronic kidney injury. In patients with renal colic because of urolithiasis, elevation in creatinine serum concentration is often observed. Nevertheless, this classical kidney function indicator lacks specificity in detecting the underlying cause of renal damage and rises late in the process of acute injury. There are various biomarkers proposed to identify and monitor the process of kidney injury. A few of them may apply to urolithiasis as well. 

### 4.2. Cystatin C

Cystatin C has definitely gained importance in diagnosing AKI and CKD. It is a low molecule (13.3 KD) removed from the bloodstream by the kidneys, and its serum levels are a more precise test of kidney function than serum creatinine levels [41]. Most studies prove that cystatin C levels are less dependent on age, gender, ethnicity, diet or muscle mass when compared to creatinine [42]. Cystatin C is a good kidney biomarker in a range of different conditions, including diabetic patients, CKD and after kidney transplantation [43].

We analyzed more recent research on cystatin C in urolithiasis but got conflicting results. 

In the study of Hughes S. et al., who compared pre-ureterorenoscopy (URS) and post-URS cystatin C levels in urolithiasis patients, no significant difference was found [23]. In 2020, Kovacevic L. et al. found significant cystatin C elevation in patients with urolithiasis [27]. We should wait for new studies on larger groups of affected participants to make a reliable conclusion on cystatin C’s role in urolithiasis.

### 4.3. Neutrophil Gelatinase-Associated Lipocalin (NGAL)

Neutrophil gelatinase-associated lipocalin NGAL is a 25 kDa protein bound to gelatinase from neutrophils. Its expression was shown in the proximal and distant tubular cells of the kidney [44]. NGAL is upregulated during the inflammatory process because it meditates cellular proliferation and differentiation and has a bacteriostatic effect [45]. It is a marker of great interest in acute tubular damage, as the expression is upregulated 2–4 h post nephrotoxic and ischemic kidney injury [46,47,48]. Evidence from several studies points out that NGAL is useful in the detection or monitoring of kidney disorders where tubules are affected [49]. In the report of Bolgeri M. et al., not only patients with obstructive uropathy but also those who had urolithiasis without blockade in urine flow had higher sNGAL and uNGAL when compared to healthy peers [50]. Some reports give evidence that the highest uNGAL levels are observed in patients with urolithiasis combined with urinary tract infection [51], which seems to be understood as this marker rises in inflammatory conditions. In the more recent study of Tasdemir M. et al. who compared, among others, uNGAL levels in patients with nephrolithiasis, it was shown that only those who had hydronephrosis (HN) had also elevation in uNGAL [15]. This observation may propose a very careful hypothesis that in patients with nephrolithiasis without HN, markers of tubular injury are not increased because urine flow is not interrupted and tubular injury is not present in contrast with those where dilation of the renal pelvis was observed together with a rise in uNGAL/cr and uNAG/cr. The biggest limitation of this observation is the small number of patients with HD that were included. Other recent findings on NGAL in stone formers are presented in the table.

### 4.4. Kidney Injury Molecule 1

Kidney Injury Molecule 1 (KIM-1) is a transmembrane protein produced by proximal tubules and is present in plasma and urine after renal injury [52]. It was detected 12–24 h post-AKI, and higher urinary values were observed in patients with ischemic acute tubular necrosis than in other conditions, including CKD, diabetic nephropathy or steroid-resistant nephrotic syndrome [53]. Several studies assessed KIM-1 in nephrolithiasis, giving conflicting results. Some researchers found that uKIM-1 was increased in patients with obstructive nephropathy [54,55]. Similarly, in the study of Fahmy et al., the elevation of uKIM-1 was clear in patients who underwent retrograde intrarenal surgery and shock wave lithotripsy because of kidney stones compared to healthy controls [56]. These findings are in contrast to Urbschat A. et al. who found no difference in uKIM-1 between participants with obstructive nephropathy and controls [57]. The reviewed results of the recent research on KIM-1 as a marker of urolithiasis are gathered in Table 1.

### 4.5. Carbohydrate Antigen 19-9

Carbohydrate Antigen 19-9 (CA 19-9) is a 36-kD glycoprotein normally expressed in different tissues starting from the gastrointestinal tract, through the bronchi or endometrium and ending in the prostate. It is best known as a marker of pancreatic and other gastrointestinal cancers [58,59,60]. Nevertheless, some investigators revealed its higher urinary levels in urinary tract obstruction [61,62,63]. Suzuki K. and Kajbafzadeh A.M. found that CA 19-9 could be a marker for kidney injury related to urinary obstruction in their research on patients with hydronephrosis [61,62]. Amini E. et al. conducted a study on people with urolithiasis and HN before and after a procedure called transurethral lithotripsy [13]. The affected group had a significant elevation before the operation, which decreased in the following measurements.

It was also noted that the duration of ureteral obstruction was correlated with serum and urinary CA 19-9 levels, which may suggest its potential as a predictive molecule for renal damage. CA 19-9 may seem to be sensitive in the detection of urine flow blockade, but it is not specific to the urinary tract, and this significantly limits its use as a marker.

### 4.6. N-Acetyl-B-D-Glucosaminidase

N-acetyl-B-D-glucosaminidase (NAG) is also one marker of tubular damage; however, in vivo studies on urolithiasis and its urinary levels were not elevated in stone formers [64]. Nevertheless, in the recent study of Xiaohong F. et al., it was found that bilateral stone formers were more endangered with CKD and had, among others, an increased urine NAG/creatinine ratio (OR 1.95; 95% CI 1.21–3.16) when compared with healthy peers [32].

### 4.7. Myeloperoxidase

Myeloperoxidase (MPO) is involved in the generation of oxygen radicals by neutrophils in inflammatory conditions [65]. It may rise in the kidney formers; however, it was not proved in an in vivo study. Hughes S. et al. compared pre- and post-URS MPO values in stone-forming participants, and no differences were observed between these two groups [23]. 

### 4.8. Markers of Inflammation

#### 4.8.1. Interleukins

Interleukins are cytokines involved mainly in inflammatory response. Most studied in the kidney disorders are listed below. Different tissues like macrophages, osteoblasts and smooth muscles in vessels produce IL-6, which can cause inflammation. It is also a myokine released by muscles in response to excessive contractions, and, in this role, it has mainly an anti-inflammatory effect by inhibition of TNF-alpha [65]. Its importance was shown in many diseases, including different cancers, obesity and severe COVID-19 infection [66]. In sepsis with AKI, an elevation of IL-6 was also noticed [67,68]. IL-8 is another potent cytokine that accelerates inflammation. It induces chemotaxis in target cells (neutrophils and other granulocytes) to make them migrate to the site of infection and then stimulates phagocytosis [66]. As a marker of inflammation, a urinary IL-8 increase was noticed in the course of pyelonephritis [69]. IL-18 can modulate innate and adaptive immunity, and dysregulation of its distribution can lead to autoimmune or inflammatory diseases. The primary site of IL-18 production is macrophages in various organs. It was found in the proximal tubular cells of the kidney as well. IL-18 seems to be the most involved cytokine in kidney disorders. It was even proposed to be the marker of early AKI as it increases 6–24 h post-starting factor. In the kidney, IL-18 is also associated with excessive urinary protein excretion and can be a marker of the progression of diabetic nephropathy [70,71]. Research on inflammatory cytokines in urolithiasis is not consistent. In the study of Memmos D. et al., who compared the effect of standard percutaneous nephrolithotomy (sPCNL) with miniaturized PCNL (mPCNL) and retrograde intrarenal surgery (RIRS) as a nephrolithiasis treatment and measured, among others, uIL-18/cr ratios at baseline and 2, 6, 24 and 48 h postoperatively in the above patients, no significant differences in its level were shown. Similarly, no between-group changes were observed for urinary IL-18/cr at 2 h and later time points postoperatively. Within particular groups, increases for IL-18/cr from baseline were noted at 2 h and progressively lower rises from time zero in all participants at 6, 24 and 48 h post-procedure. No significant difference in this marker level was noticed in AKI or other complications [35]. Similarly, in the study of Kandur Y. et al., who assessed urinary IL-18/cr in 40 pediatric patients diagnosed with nephrolithiasis (NL), 23 patients with hypercalciuria (HC) and 20 healthy controls, no significant differences between patient and control groups regarding urinary IL-18/cr were observed [12]. Kusumi K. et al. observed that uIL-13 was significantly increased in lithiasis participants, while IL-17A was elevated in the urine of controls [21]. More results of the most recent research on urinary interleukins as markers of urolithiasis are in Table 1. They seem to be conflicting and need further observations on larger and homogenous groups of subjects. 

#### 4.8.2. Tumor Necrosis Factor—α

Tumor necrosis factor—α (TNF-α) is both an adipokine and cytokine. As a cytokine, it is used for cell signaling. Macrophages detecting an infection release TNF to alert other immune system cells and start an inflammatory response. TNF-α regulates cell proliferation, differentiation and apoptotic death and may be used in the detection of various renal disorders [72]. Hughes S. et al. observed that TNF-α levels significantly increased in lithiasis patients undergoing SWL, peaking at 30 min post-SWL [25].

#### 4.8.3. Monocyte Chemoattractant Protein 1

Monocyte chemoattractant protein 1 (MCP-1) is an inflammatory chemokine produced by mononuclear and intrinsic cells in the kidney to activate and recruit monocytes [73]. Its upregulation is the response to various damaging factors. Studies on several kidney disorders demonstrated its potential as a biomarker. Lupus nephritis severity correlated with urinary MCP-1 (uMCP-1) levels in pediatric patients. Similarly, MCP-1 was higher in patients with chronic kidney disease (CKD) or autosomal recessive polycystic kidney disease (ARPKD) when compared to healthy controls [74]. In urolithiasis, MCP-1 may find its place as well. In the research of Umekawa T. et al., exposure of cultured renal epithelial cells (from a rat renal proximal tubular cell line) to CaOx crystals resulted in higher expression of MCP-1 mRNA and an increased level of the protein [75]. A recent study by Wang X. et al. found that primary hyperoxaluria patients who form stones have high levels of MCP-1 in their urine, which may indicate ongoing crystallization. Other studies on MCP-1 are gathered in Table 1.

#### 4.8.4. C-Reactive Protein, Procalcitonin

CRP was first identified by Tillet and Francis in 1930. They found that it can make streptococcus pneumoniae C-polysaccharide precipitate. CRP is synthesized in the liver as a fast response to inflammation and decreases rapidly after its resolution [76]. Similarly, procalcitonin (PCT) concentration rises as a reaction to a pro-inflammatory stimulus, especially of bacterial origin. Both CRP and PCT are commercially used in blood laboratory tests to detect severe inflammatory diseases. In several studies on urolithiasis, it was shown that elevation in CRP and PCT occurs, especially when stones cause obstruction leading to inflammation of the surrounding tissue [77]. Choosing the best treatment option for ureter obstruction depends on different factors, including the size of a stone and its location. According to The European Association of Urology, medical expulsive therapy (MET) with the use of an alpha blocker should be started in patients with renal colic and distal ureteric stones less than 5 mm, whose symptoms are controlled [78]. Classical inflammatory markers can also support the decision-making process. Observations of Cilesiz et al. show that stone formers who failed MET had higher values of serum PCT than those who spontaneously passed the stone [26]. Similar observations considering serum CRP were demonstrated earlier by Özcan C. et al. [79] as well as Aldaqadossi H. et al. [80] and Jain with colleges [81]. They gave cut-off values of CRP as a predictor of spontaneous stone passage ranging from 0.506 to 21.9 and 4.1 mg/L, respectively. Further research is needed to determine the optimal cut-off level for classical inflammatory indicators in urolithiasis resulting in ureteral obstruction, despite the certainty of their role.

### 4.9. Macrophage Inflammatory Protein 1beta 

Macrophage Inflammatory Protein 1beta (MIP1β) is a chemokine starting recruitment of the immune cells responsible for innate and adaptive immune activity. It is involved in the process of inflammatory response during infection [82]. As another inflammatory molecule, it may be present in stone-induced kidney injury. In the study of Kusumi K. et al., urinary MIP1β levels were significantly elevated in stone-forming adolescents compared to healthy controls [21].

### 4.10. Other Urinary Proteins

#### 4.10.1. Osteopontin

Osteopontin (OPN) is a phosphorylated protein that plays an essential role in bone mineralization [83]. Most probably, it is also involved in the process of inflammation, cell survival and leukocyte recruitment [84]. It has a wide tissue distribution associated with abnormal calcification including an organic matrix of the kidney stones. OPN is produced in the kidney and found in human urine. It probably acts as one of the urolithiasis inhibitors by preventing the formation of CaOx and further adhesion of crystals to renal epithelial cells [85,86]. Nevertheless, some research on animals gives exactly the opposite conclusions [87,88]. Some authors observed that OPN may increase the risk of CaOx urolithiasis by progression of renal tubular cell damage [87,88].

In the recent study by Icer et al., who compared urinary excretion of OPN between patients with urolithiasis and healthy controls, it was shown that affected participants had significantly lower levels of OPN than unaffected peers [16]. They concluded that low urinary OPN levels were correlated with a higher risk of urolithiasis. More studies on OPN are presented in Table 1.

#### 4.10.2. Nephrocalcin

Nephrocalcin (NC) is one of the most studied molecules, taking part in the process of stone formation as an inhibitor of calcium oxalate (CaOx) crystallization. It was first described in 1978 by Nakagawa et al. as an unidentified acidic polypeptide [89]. During the next ten years, several studies showed its inhibitory effect on CaOx crystallization, and in 1987, it was isolated from the urine and named nephrocalcin [89,90,91,92,93]. This glycoprotein is in the proximal tubule and thick ascending limb of the Henle’s loop [94]. It has many polymeric forms and at least four isoforms: NC-A, NC-B, NC-C and NC-D. The risk of CaOx crystallization and nephrolithiasis depends on the proportion of the isoforms in the urine [95]. Those who are more likely to develop kidney stones excrete greater proportions of NC-C and -D than NC-A and -B [96]. Noyan et al. assessed the NC-PreA/cr ratio in the urine of 41 stone-forming children and 25 matched healthy controls. It appeared to be significantly higher in affected participants [97].

### 4.11. Bikunin

Another protein that slows CaOx crystallization is bikunin. It is a small chondroitin sulfate proteoglycan joined with a single glycosaminoglycan chain localized in the proximal tubule and the thin descending part of the Henle loop [98]. It is a potent inhibitor of CaOx crystal nucleation and aggregation mostly in healthy humans, whereas in the presence of urolithiasis, its preventing role is limited [99,100]. The existing literature on bikunin’s role gives conflicting results. Higher levels of bikunin were found in children with urolithiasis while in another study on adults, those affected by kidney stones had 50% lower urinary concentration when compared to healthy peers [101,102].

### 4.12. Calgranulins

Calgranulins, otherwise S100 proteins, are a family of calcium-binding molecules present in the cytosol. Some of them, including S100A8 (calgranulin A) and S100A9 (calgranulin B) have been classified as danger-associated molecular patterns of endogenous origin—alarmins, a group of molecules released as inflammatory signal mediators after cell death [103]. Normally, calgranulins A and B are produced mainly by neutrophils and monocytes, as well as dendritic cells, while in other cell types, they appear after the activating signal [104,105]. Momohara C. et al. found that calgranulins inhibit the crystallization, aggregation and adhesion of CaOx monocrystals to the endothelium, while Mushtaq S. et al. found that they promoted crystal aggregation [106,107].

In the more recent study of Jung K. et al. in children with urolithiasis, no significant differences were observed between the study and control groups [17].

### 4.13. Matrix Gla Protein

Matrix Gla protein (MGP) was identified in the bone matrix and then in other tissues, including vascular [108]. Although it is suspected to have an inhibitory effect on calcification, the recent study on stone formers does not confirm this hypothesis [24,108].

### 4.14. Tamm–Horsfall Protein

Tamm–Horsfall protein (THP), known as uromodulin, is one of the most extensively investigated macromolecules in nephrolithiasis. It is found in the urine of all placental invertebrates as a polymer with a molecular weight of up to several million Da [109,110]. It is involved in the pathogenesis of nephrolithiasis and tubule interstitial nephritis [111]. The studies on the particular role of THP in stone formation give conflicting answers. Some point out that it is an inhibitor of crystallization [112,113,114,115], whereas others point out its promoting role [112,113,114,115,116,117,118]. Perhaps its action depends on the concentration of this protein and other solutes [119]. In a recent study by Nonnan C. et al., THP was found to inhibit crystallization by binding calcium ions in a concentration-dependent manner [120].

### 4.15. Urinary Prothrombin Fragment—1

Urinary prothrombin fragment—1 (UPTF-1) is a part of thrombin. There are both in vitro and in vivo studies indicating its inhibitory effect on crystallization. Epidemiological observations confirm a bigger incidence of stone formation in people who had lower UPTF—1 [121,122]. 

## 5. Conclusions

In this review, we explored the most discussed proteins that are associated with the process of stone formation. Recent studies on proteins involved in stone formation are conflicting and inconclusive, making it difficult for clinicians to choose the best treatment.

The above might have a few explanations. First, urolithiasis, although related to idiopathic hypercalciuria in about 70–80% of cases, is not one homogenous disorder. Kidney stones may be composed of different minerals. What is more, there are various co-factors promoting their formation. The patients studied were different in age, kidney function, urinary tract defects and other health issues including medications.

What is more, the number of participants was usually low, below 100. There is one more important matter that should be taken into consideration while reviewing the existing literature and projecting future research. In the findings of Sheng X. et al., who noticed that CaOx monohydrate adhesion to the epithelium depended on the presence of protein carboxyl groups, it was pointed out that the inhibitory effect of macromolecules was related not only to their urinary levels but also the histochemical features [123]. It is important to examine not only their quantity but also quality, which was usually missed in the recent research.

Nevertheless, some proteins like NGAL, KIM-1, OPN or THP seem to be more attractive as proposed biomarkers. More reliable conclusions can be drawn in the future by conducting research on larger, similar populations with urolithiasis and using validated proteomic methods.

## Figures and Tables

**Table 1:** Results of the studies from the last seven years on new protein kidney biomarkers in urolithiasis.

Year of Publication	Author and Title	Study Design	Results
2016	**Kandur Y. et al.** [12] Evaluation of urinary KIM-1, NGAL, and IL-18 levels in determining early renal injury in pediatric cases with hypercalciuria and/or renal calculi.	Urinary NGAL, KIM-1 and IL-18 levels were measured in 40 pediatric patients diagnosed with nephrolithiasis (NL), 23 patients with hypercalciuria (HC) and 20 healthy controls.	A significant difference was found between patient groups (NL and HC) and healthy children regarding urinary NGAL/cr ratio (*p* < 0.001). There were no significant differences between patient and control groups regarding urinary IL-18/cr and KIM-1/cr ratios.
2016	**Amini E. et al**. [13] The role of serum and urinary carbohydrate antigen 19-9 in predicting renal injury associated with ureteral stone.	This study was designed to evaluate the role of urinary and serum carbohydrate antigen 19-9 (uCA19-9 and sCA19-9) as a biomarker in the assessment of patients with ureteral stones. A total of 38 patients with ureteral stones and hydronephrosis who underwent transurethral lithotripsy (TUL) (Group A) and 24 age-matched healthy peers (Group B) were evaluated in this study. Urinary and serum CA19-9 concentrations were measured in group A before TUL and 4 and then 8 weeks following the operation; sCA-19-9 and uCA19-9 concentrations were also measured in group B participants.	Median concentration of uCA19-9 and sCA19-9 was 34.0 and 15.0 kU/L in group A patients and 16.1 and 5.3 kU/L in group B, respectively (*p* < 0.001). Medians of CA19-9 concentration in urine and serum reduced to 12.5 and 4.5 kU/L 8 weeks after TUL (*p* < 0.001). Following successful TUL and hydronephrosis resolution, a significant decline was detected in serum and urinary CA19-9. The duration of ureteral obstruction was associated with serum and urinary CA19-9 concentrations, suggesting the potential role of this marker in predicting renal damage associated with urinary tract obstruction and determining the timing of interventions.
2017	**Venkatesan, S et al.** [14] Association between vitamin D, parathyroid hormone and inflammatory markers in urolithiasis patients	It was a cross-sectional study. A total of 41 confirmed renal calculi patients and 41 age and sex-matched controls were recruited. Patients with malignancies, hyperparathyroidism or chronic disease and patients taking vitamin D supplementations were excluded. Serum levels of 25(OH) vitamin D, i-PTH, hs-CRP, IL-6, calcium and phosphorous, 24 h urine levels of calcium and phosphorus were estimated.	There was a significant difference in the serum levels of 25(OH) vitamin D (12.26 vs. 19.61 ng/mL), i-PTH (75.5 vs. 33.5 pg/mL), hsCRP (5117.05 vs. 1721.87 ng/mL), IL-6 (13.49 vs. 1.47 pg/mL), calcium (11.5 vs. 9.4 mg/dL), urinary calcium (370.5 vs. 342 mg/d) and phosphorous levels (1172 vs. 1432 mg/d) between the cases and the control. There was a negative correlation between the levels of i-PTH and vitamin D (r = −0.765) and positive correlation between i-PTH and hsCRP, IL-6, serum calcium and urine calcium (r = 0.353, 0.340, 0.522, 0.501, respectively).
2018	**Taşdemir M. et al**. [15] Urinary biomarkers in the early detection and follow-up of tubular injury in childhood urolithiasis.	Seventy children (thirty-six girls, mean age: 7.3 ± 5.0 years (0.5–18.2)) with urolithiasis/microlithiasis were included. Anthropometric data, urinary symptoms, family history and diagnostic studies were recorded. Urine samples were analyzed for metabolic risk factors (urinary calcium, uric acid, oxalate, citrate, cystine, magnesium and creatinine excretion), and the urinary KIM-1 (uKIM-1), NAG (uNAG) and NGAL (uNGAL) levels were measured 3 times in 6-month intervals.	UKIM-1/Cr, uNAG/Cr and uNGAL/Cr ratios were not significantly different between patients and controls. Furthermore, no significant changes in their excretion were shown during follow-up. Only uNAG/Cr and uNGAL/Cr ratios were significantly increased in patients with hydronephrosis (n = 6, *p* = 0.031 and 0.023, respectively).
2018	**Icer M. A. et al.** [16] Can urine osteopontin levels, which may be correlated with nutrition intake and body composition, be used as a new biomarker in the diagnosis of nephrolithiasis?	This study was conducted on 88 volunteers, including 44 healthy individuals and 44 patients diagnosed with nephrolithiasis and aged between 20 and 65 years. Some serum parameters and urinary osteopontin (uOPN) levels of the individuals were analyzed. Several anthropometric measurements of the individuals were taken, and their body mass index was calculated. Their 24 h dietary recall and water intakes were recorded, and the participants completed a food-frequency questionnaire for the evaluation of their nutritional status.	UOPN (ng/mL) levels of patients were lower than those of the control group (*p* < 0.05). Dietary energy, carbohydrate, poly-unsaturated fatty acid (PUFA) and n-6 fatty acid intakes and uOPN levels of male patients were positively correlated (*p* < 0.05). There was a negative correlation between uOPN (ng/mL) and serum creatinine (mg/dL) levels of female patients (*p* < 0.05). Body weight, waist circumference, hip circumference and body muscle mass values of healthy males were positively correlated with their uOPN levels (*p* < 0.05).
2018	**Jung K. et al.** [17] Assessment of cross-correlations between selected macromolecules in urine of children with idiopathic hypercalciuria.	The study was designed to assess protein macromolecules uromodulin, osteopontin, calgranulin and bikunin in a fresh morning urine sample in children with nephrolithiasis in the course of idiopathic hypercalciuria. The study included 57 affected subjects aged 12 months–18 years; control group comprised 33 healthy peers.	In the urine of lithiasis group, significantly decreased ostepontin excretion and significantly increased bikunin excretion were shown. Calgranulin excretion was increased; however, it missed statistical significance. There was also a significant positive correlation between uromodulin and bikunin in both groups.
2019	**Chirackal R.S. et al.** [18] Urinary extracellular vesicle-associated **MCP-1** and **NGAL** derived from specific nephron segments differ between calcium oxalate stone formers and controls.	The study was designed to test the hypothesis that extracellular vesicles (EVs) containing potential biomarkers for inflammation (monocyte chemoattractant protein, MCP-1), kidney epithelium injury (neutrophil gelatinase-associated lipocalin, NGAL) and abnormal calcification (osteopontin, OPN) might reflect intrarenal stone formation process. A total of 64 calcium formers (CFs) and 40 age- and sex-matched healthy peers were included. Urolithiasis participants were divided into 2 subgroups: with low (<5%) and high papillary surface area that reflect the respective amount of Randall plaques (precursors of crystallization). EVs carrying MCP-1, NGAL and OPN were assessed in the urine.	The number of EVs carrying MCP-1 and NGAL was significantly lower in CF participants compared with healthy group, but it did not differ between patients with low and high papillary surface area. The number of EVs with OPN did not differ between any groups.
2019	**Shah T.T. et al.** [19] Factors associated with spontaneous stone passage in a contemporary cohort of patients presenting with acute ureteric colic: results from the Multi-centre cohort study evaluating the role of Inflammatory Markers In patients presenting with acute ureteric Colic (MIMIC) study	The study included 4170 patients with acute ureteral colic and a single ureteral stone (confirmed by CT scan). The researchers assessed the association between white blood cell (WBC) count and other routinely collected inflammatory and clinical markers, including stone size, stone location and use of medical excretory therapy (MET), with spontaneous stone passage (SSP). The main outcome measure was spontaneous stone passage (SSP), defined as no need for intervention to facilitate stone passage (SP).	Results of multifactorial analysis showed that neither WBC, neutrophil count nor C-reactive protein (CRP) predicted SSP, with corrected odds ratios (ORs) of 0.97 (95% confidence interval (CI) 0.91–1.04, *p* = 0.38), 1.06 (95% CI 0.99–1.13, *p* = 0.1) and 1.00 (95% CI 0.99–1.00, *p* = 0.17).
2019	**Okada A. et al.** [20] Identification of new urinary risk markers for urinary stones using a logistic model and multinomial logit model	Male participants (age, 20–79 years) were divided into three groups: a control group (n = 48) with no history of stones and two stone groups with calcium oxalate stone experience (first-time group, n = 22; recurring group, n = 40). Using 25 -µL spot urine samples, they determined the concentrations of 18 candidate urinary proteins, using multiplex analysis on a MagPix (R) system.	In univariate logistic regression models, classifying the control and first-time groups, interleukin (IL)-1a and IL-4 were independent factors, with significantly high areas under the receiver operating characteristic curve (1.00 and 0.87, respectively, *p* < 0.01 for both). The multivariate models with IL-4 and granulocyte-macrophage colony-stimulating factor (GM-CSF) showed higher areas under the receiver operating characteristic curve (0.93) compared to that for the univariate model with IL-4. In the classification of control, first-time and recurrence groups, accuracy was the highest for the multinomial logit model with IL-4, GM-CSF, IL-1b, IL-10 and urinary magnesium (concordance rate 82.6%).
2019	**Kusumi K. et al.** [21] Adolescents with urinary stones have elevated urine levels of inflammatory mediators.	The main objective of this study was to compare urinary inflammatory markers in stone-forming children versus healthy matched controls. The urine samples were collected from 12 adolescents with urolithiasis and 15 controls. The levels of 30 urinary cytokines were assessed with the use of a Mesoscale 3-0-Plex Human Cytokine panel and normalized to urine creatinine.	Among others, MIP1β and IL13 were significantly increased in affected participants. Interleukin 17A was elevated in the urine of controls.
2020	**Wang X. et al.** [22] Urinary monocyte chemoattractant protein 1 associated with calcium oxalate crystallization in patients with primary hyperoxaluria.	A total of 30 patients with primary hyperoxaluria and CaOx crystals and 47 healthy peers were included. In the urine samples of study participants, a panel of biomarkers reflecting different nephron sites and potential mechanisms of injury was assessed: clusterin, neutrophil gelatinase-associated lipocalin (NGAL), 8-isoprostane (8-IP), Monocyte Chemoattractant Protein -1 (MCP-1), Liver-fatty acid binding protein (L-FABP), heart-type fatty acid binding protein (H-FABP) and osteopontin (OPN).	After adjustment for age-, sex- and eGFR, a higher urinary MCP-1 concentration and MCP-1/creatinine ratio was positively correlated with CaOx supersaturation. Higher urinary NGAL and NGAL/creatinine, as well as urinary OPN and OPN/creatinine, were related to higher eGFR. It seems that higher urinary MCP-1 might reflect ongoing collecting tubule crystallization, while greater urinary NGAL and OPN may reflect preservation of kidney mass and function.
2020	**Hughes S.F. et al.** [23] The role of specific biomarkers, as predictors of post-operative complications following flexible ureterorenoscopy (FURS), for the treatment of kidney stones: a single-centre observational clinical pilot-study in 37 patients.	A total of 37 patients (24 men, 13 women) who underwent planned FURS for the treatment of kidney stones were included in the study. They collected blood samples from each patient: preoperatively and at 30 min, 2 h and 4 h postoperatively. Changes in renal biomarkers (NGAL, Cystatin-C) and infectious biomarkers (MPO, PCT) were assessed.	There were postoperative complications in 4 patients (3—UTIs with urinary retention, 1—urosepsis). NGAL concentrations increased markedly after FURS (*p* = 0.034). There were no statistically significant differences in cystatin C, MPO and PCT concentrations. The study’s limiting factors were the small number of recruited subjects and the reduction in blood sampling 4 h after the procedure.
2020	**Castiglione V. et al.** [24] Evaluation of inactive Matrix-Gla-Protein (MGP) as a biomarker for incident and recurrent kidney stones.	They measured serum dpucMGP in incident symptomatic kidney stone formers and non-stone formers at a baseline visit. Symptomatic stone recurrence was assessed in the stone formers over a 5-year period. The association of dpucMGP with incident or recurrent kidney stones was assessed with and without adjustment for clinical, blood and urine characteristics.	There was no significant difference in serum dpucMGP level between 498 stone formers and 395 non-stone formers (510 vs. 501 pmol/L; *p* = 0.66). In a multivariable model, adjusting for clinical, blood and urine chemistries, higher MGP was associated with lower risk of stone formation (OR = 0.674, 95% CI 0.522–0.870), contrary to previous reports. Among 375 stone formers with 5 years of follow-up, 79 (21%) had symptomatic recurrence. No difference in serum dpucMGP was evident in recurrent versus non-recurrent stone formers (482 vs. 502 pmol/L; *p* = 0.26). Serum dpucMGP was correlated with cystatin C levels in non-stone formers, incident stone formers and recurrent stone formers (r > 0.3, *p* < 0.0001).
2020	**Hughes S.F. et al.** [25] Shock wave lithotripsy, for the treatment of kidney stones, results in changes to routine blood tests and novel biomarkers: a prospective clinical pilot-study.	The researchers recruited 12 patients after SWL for single unilateral kidney stones. Blood samples were collected from the patients (8 males and 4 females) aged between 31 and 72 years (median 43 years) of blood before surgery, at 30, 120 and 240 min after surgery. Concentrations of NGAL, IL-18, IL-6, TNF-α, IL-10 and IL-8 were measured using ELISA kits.	NGAL concentrations increased significantly, reaching peak values 30 min after treatment (*p* = 0.033). IL-6 presented a statistically significant increase from the preoperative period to 4 h postoperatively (*p* < 0.001), while TNF-α increased significantly, peaking at 30 min. While IL-18 concentrations increased, these alterations were not significant (*p* = 0.116). No significant alterations in IL-10 and IL-8 concentrations were observed after SWL (*p* > 0.05).
2020	**Cilesiz N.C. et al.** [26] Can serum procalcitonin levels be useful in predicting spontaneous ureteral stone passage?	The purpose of the study was to assess predicting role of procalcitonin (PCT) in blood of participants with a single distal ureteral stone (size 5–10 mm) who did not have any indications for urological intervention and healthy controls. Blood was tested for WBC (white blood cell), c-reactive protein (CRP), SED (sedimentation), MPV (mean platelet volume), NLR (neutrophil-to-lymphocyte ratio) and serum procalcitonin (PCT) concentration, and urinalysis was performed. The samples were taken in 2-week intervals for 1 month or until the intervention. The group of patients was divided into participants with complete stone passage (SP(+)) and failure (SP(−))—stone was still in the ureter. The patients underwent MET with tamsulosin 0.4 mg/day, diclofenac sodium 75 mg/day and hydration—at least 3 L/day. During follow-up, kidney, ureter and bladder (KUB) plain films, ultrasonography (USG) and abdominal CT scans were performed.	It appeared that SP(−) participants had PCT concentration significantly higher (207 ± 145.1 pg/mL) than in the SP(+) group (132.7 ± 28.1 pg/mL) (*p* = 0.000). ROC curve analysis revealed that 160 pg/mL (86.7% sensitivity, 70.8% specificity, *p* < 0.001; AUC: 0.788 95% CI (0.658–0.917)) was the optimal cut-off value for PCT. The leukocyturia of the SP(−) group was significantly higher than in the SP(+) group (*p* = 0.004). In logistic regression analysis, a significant efficacy of PCT and leukocyturia was observed in the univariate analysis on spontaneous passage. In the multivariate analysis, significant independent activity was observed with PCT (*p* < 0.05).
2020	**Kovacevic L. et al**. [27] Cystatin C, Neutrophil Gelatinase-associated Lipocalin, and Lysozyme C: Urinary Biomarkers for Detection of Early Kidney Dysfunction in Children With Urolithiasis.	A pilot, prospective study of pediatric patients with urolithiasis (RS) and their age- and gender-matched controls (HC). Preliminary screening was performed by a proteomic comparison of urine samples collected from RS and HC, using liquid chromatography-mass spectrometry.	Proteomic evaluation identified 3 highly interesting proteins, cystatin C (CYTC), neutrophil gelatinase-associated lipocalin (NGAL) and lysozyme C, which were significantly over-represented in the RS group compared with HC. Urinary CYTC and NGAL had significantly elevated levels, while lysozyme C had almost significantly elevated levels in the RS group (N = 24) compared to the control group (N = 13). Urinary CYTC levels were increased in both hypercalciuria (N = 14) and hypocitraturia (N = 10) compared to HC (*p* < 0.05).
2021	**Taiguo Q. et al**. [28] The predictive and diagnostic ability of IL-6 for postoperative urosepsis in patients undergoing percutaneous nephrolithotomy.	The aim of this study was assessment of the predictive and diagnostic role of IL-6 for postoperative urosepsis in patients undergoing percutaneous nephrolithotomy (PCNL). Ninety patients undergoing PCNL between April 2019 and September 2019 were studied. A total of 16 patients progressed to urosepsis (EXP1 group, n = 16), and 74 patients did not (CON group, n = 74). Twenty-five patients who progressed to postoperative urosepsis without receiving the test of IL-6 between March 2018 and March 2019 were also enrolled (EXP2 group, n = 25); demographic and perioperative data were compared between all groups.	Compared with CON group, EXP1 group showed higher serum levels of IL-6 (*p* < 0.001) and neutrophil (*p* < 0.001) at postoperative hour two; higher serum levels of IL-6 (*p* < 0.001), procalcitonin (PCT) (*p* < 0.05), white blood cell (WBC) (*p* < 0.05) and neutrophil (*p* < 0.001) on postoperative day one; and higher serum levels of PCT (*p* < 0.05) and WBC (*p* < 0.05) on postoperative day three. ROC curves showed IL-6 (AUC = 1.000) at postoperative hour two and PCT (AUC = 0.954) on postoperative day three. Compared with EXP2 group, EXP1 group showed shorter time to intervene (*p* < 0.001), a shorter postoperative hospital stay (*p* < 0.001) and a lower incidence rate of severe urosepsis (*p* < 0.05). The main conclusion was the possible predictive values of IL6 as an early diagnostic marker for postoperative urosepsis in patients after PCNL at postoperative hour two and postoperative day one.
2021	**Ramasamy V. et al.** [29] Role of inflammatory markers and their trends in predicting the outcome of medical expulsive therapy for distal ureteric calculus.	The purpose of this study was to investigate the role and trends of the inflammatory markers C-reactive protein (CRP), white blood cell (WC) count and neutrophil (NP) percentage in predicting the outcome of medical excretory therapy (MET). 192 patients with distal ureteral stones > 5 mm in size were included in the study from April 2017 to March 2018. CRP, WC and NP were measured on days 1, 7 and 14 MET and then analyzed.	CRP, WC and NP mean and size on all days in patients who did not pass stones were significantly higher compared to those who passed stones. The area under the curve in the ROC analysis showed values of 0.798 (*p* = 0.001) for CRP, with a cut-off value of 1.35 mg/dL. Multifactorial analysis showed a significant association of higher CRP levels > 1.35 mg/dL and stone size > 7 mm with MET failure. A decreasing trend in CRP was observed in both groups, but values were higher in those who had not passed stones. WC and NP decreased only in those with stones.
2021	**Milisic E. et al.** [30] Urinary neutrophil gelatinase—associated lipocalin level as a biomarker of acute kidney injury following extracorporeal shock lithotripsy.	The aim of this research was to evaluate the severity of the kidney tissue response to extracorporeal shock wave lithotripsy (ESWL) injury by measuring the urinary neutrophil gelatinase-associated lipocalin (uNGAL) as an indicator of acute kidney injury (AKI) in the early phase. The study included 62 patients with nephrolithiasis undergoing single ESWL therapy. UNGAL level was measured before the procedure and 6 h and 12 h after it.	The median uNGAL level increased by 126% 6 h post-ESWL (*p* < 0.001) with further growth up to 583.7% after 12 h when compared to pre-treatment level. The median estimated glomerular filtration rate (eGFR) dropped by 15.3% 12 h post-intervention but increased by 5% in the period of 7 days to 3 months after. uNGAL level was significantly negatively correlated with eGFR 12 h, 7 days and 3 months after ESWL. The sensitivity of uNGAL 12 h after ESWL was 60.6% and specificity 5% with a positive predictive value of 74% and negative predictive value of 61.7%. UNGAL had the highest predictive value of AKI 12 h after the ESWL treatment.
2021	**Wymer, K.M. et al.** [31] A Serum C-Reactive Protein and Procalcitonin-Based Risk Score to Predict Urinary Infection in Patients with Obstructive Urolithiasis Undergoing Decompression	A retrospective review of patients presenting to the emergency room from December 2017 to February 2019, who underwent upper urinary tract decompression due to concerns about infection for urolithiasis, was conducted. More than 30 clinical parameters were evaluated, and a composite risk score was created. Univariate and multivariate forward stepwise regression analyses were performed to identify predictors of true urinary tract infection (UTI).	A total of 98 patients fulfilled the inclusion criteria, of which true UTI was identified in 50 (51%). The standard model of serum white blood cell count > 15 or temperature > 38 degrees C had an area under the curve (AUC) of only 0.67 to predict UTI. A multivariable-regression-based 4-point risk score (1 point for each of the following: positive urine Gram stain, perinephric fat bands on CT, serum CRP > 21.95 and serum procalcitonin > 0.36) had an AUC of 0.91 to predict UTI. Individually, these components had AUCs of 0.68, 0.68, 0.80 and 0.77, respectively. The odds of confirming UTI were 8%, 11%, 68% and 100% for risk scores of 0, 1, 2 and 3 to 4, respectively (*p* < 0.001).
2022	**Xiaohong F. et al.** [32] Metabolic Differences between Unilateral and Bilateral Renal Stones and Their Association with Markers of Kidney Injury.	It was a cross-sectional study of 10,281 participants in rural China in 2014. All the subjects underwent renal ultrasound to detect urinary stones; stone formers were divided into groups with unilateral or bilateral renal stones by ultrasound examinations. CKD was defined as a decreased estimated glomerular filtration rate (eGFR, <60 mL/minute/1.73 m^2^) and/or albuminuria (albumin-to-creatinine ratio ≥ 30 mg/gm). Increased urine NAG and α1-MG levels were defined as their values above the 75th percentile of the sample distribution.	Among all the participants, 4.9% (507) had unilateral renal stones, and 0.7% (75) had bilateral renal stones. The proportion of CKD in the non-stone, unilateral and bilateral renal stone formers was 11.0%, 19.2% and 29.7%, respectively (*p* for trend < 0.001). Individuals with bilateral renal stones had the highest proportion of metabolic components, such as elevated blood pressure and serum glucose. In multivariate analyses after adjustment for multiple confounders, bilateral renal stones were significantly associated with an increased risk of decreased eGFR (OR 3.38; 95% CI 1.05–10.90), albuminuria (OR 3.01; 95% CI 1.76–5.13), CKD (OR 3.18; 95% CI 1.88–5.36), increased urine NAG/creatinine ratio (OR 1.95; 95% CI 1.21–3.16) and α1-MG/creatinine ratio levels (OR 2.54; 95% CI 1.56–4.12) compared with the lack of stones.
2022	**Noonin C. et al.** [33] Systematic analysis of modulating activities of native human urinary Tamm-Horsfall protein on calcium oxalate crystallization, growth, aggregation, crystal-cell adhesion and invasion through extracellular matrix.	This research was planned to clarify the roles of native urinary Tamm–Horsfall protein (THP) in CaOx monohydrate stone formation. In the study, 24 h urine specimens from 10 male patients with idiopathic nephrolithiasis without well-known metabolic risk factors were collected. At least 50% of stones were composed of CaOx. As controls, 10 men and 10 women were assessed without personal or family history of kidney stones. THP was purified from the urine by adsorption methods, and its effects on stone formation, crystal growth, aggregation, cell adhesion and further invasion through extracellular matrix were examined.	Analyses revealed that THP concentration-dependently (0.4–40 µg/mL) reduced CaOx monohydrate crystal size but without effect on their mass during initial crystallization. What is more, THP concentration-dependently inhibited CaOx crystal growth, aggregation and further adhesion, but it did not prevent crystal invasion through the extracellular matrix. It was found that THP has two large calcium binding domains and three small oxalate binding domains; however, in immunofluorescence, it appeared that THP binds only calcium ions with high affinity.
2022	**Kovacevic L. et al**. [34] Proteomic analysis of inhibitory protein profiles in the urine of children with nephrolithiasis: implication for disease prevention.	This is a prospective, controlled, pilot study of pooled urine from RS (N = 30, 24 females, mean age 12.95 ± 4.03 years) versus age- and gender-matched HC, using liquid chromatography-mass spectrometry. The criteria for protein selection were (1) patient/control abundance ratio of <0.5 and (2) ≤0.05 *p*-value for Fisher’s exact test. Results were confirmed by ELISA testing in individual samples.	A total of 67 proteins were downregulated in RS group, and 17 of those were significantly different compared to controls. Of those seventeen proteins, five (two actins, annexin A5, keratin 6B and serpin B4) were completely absent in the urine of stone patients but were found in controls. The remaining twelve proteins were significantly less abundant in the patients’ urine compared to healthy controls. Protein–protein interaction modeling of significant proteins identified syndecan-1 as the key node, a protein associated with adhesion pathways. ELISA analysis by subgroups showed statistically significant difference in the urinary excretion of osteopontin (5.1 ± 3.22 ng/mg creatinine vs. 14.1 ± 9.5 ng/mg creatinine, *p* = 0.046) between stone patients with hypocitraturia and controls. Urinary osteopontin concentration was positively correlated with urinary citrate excretion (r = 0.417, *p* = 0.03).
2023	**Memmos D. et al.** [35] The effect of standard percutaneous nephrolithotomy, miniaturized percutaneous nephrolithotomy and retrograde intrarenal surgery on biomarkers of renal injury: a randomized clinical trial.	The study was designed to compare the effect of standard percutaneous nephrolithotomy (sPCNL) with miniaturized PCNL (mPCNL) and retrograde intrarenal surgery (RIRS) as a nephrolithiasis treatment and measure urinary ratios: NGAL/creatinine (uNGAL/cr), KIM-1/creatinine (uKIM-1/cr) and Interleukin-18/creatinine (uIL-18/cr) at baseline and 2, 6, 24 and 48 h postoperatively.	No significant differences were shown in uNGAL/cr changes between sPGNL and mPGNL and RIRS. Similarly, no between-group changes were observed for urinary ratio KIM-1 and IL-18 at 2 h and all biomarkers at any time point postoperatively. Within particular groups, increases from baseline were noted only for uNGAL/cr, uKIM-1/cr and uIL-18/cr at 2 h, and progressively lower rises from time zero were in all participants for uKIM-1/cr and uIL-18/cr at 6, 24 and 48 h post-procedure. No significant differences in these indicators were noticed in AKI or other complications.
2023	**Savin Z. et al.** [36] The role of serum and urinary markers in predicting obstructing ureteral stones and reducing unjustified non-contrast computerized tomographic scans in emergency departments.	All emergency department (ED) patients with severe pain suggestive of obstructive urolithiasis (OU) and assessed by non-contrast computed tomography (NCCT) were included in the study. Serum white blood cell (WBC), C-reactive protein (CRP) and creatinine (Cr) levels and urine results were analyzed for association with OU, and rates of unsubstantiated NCCT scans were calculated.	NCCT diagnosed OU in 54% of patients. Median WBC, CRP and Cr values were 9100/ µL, 4.3 mg/L and 1 mg/dL, respectively. The most accurate thresholds for predicting OU were WBC = 10 000/mu L and Cr = 0.95 mg/dL (calculated using ROC curves). Only WBC >= 10 000/mu L (OR = 3.7, 95% CI 1.6–8.3, *p* = 0.002) and Cr >= 0.95 mg/dL (OR = 5, 95% CI 2.3–11, *p* < 0.001) were associated with OU. The positive predictive value and the specificity for detecting OU in patients with a total WBC count >= 10 000 and Cr >= 0.95 were 83% and 89%, respectively. In patients with negative serum marker criteria, significantly more unreasonable NCCT tests were performed (*p* = 0.03). The negative predictive value of serum criteria for a justified NCCT test was 81%.

## Data Availability

Data are contained within the article.

## Abbreviations

AKI	Acute Kidney Injury
CKD	Chronic Kidney Disease
CaOx	Calcium Oxalate
eGFR	Estimated Glomerular Filtration Rate
HN	Hydronephrosis
hs-CRP	Highly Sensitive C-reactive protein
IL	Interleukin
KIM-1	Kidney Injury Molecule-1
L-FABP	Liver-type Fatty Acid-Binding Protein
MCP-1	Monocyte Chemotactic Protein-1
MET	Medical Expulsion Therapy
MIP1β	Macrophage Inflammatory Protein 1beta
MGP	Matrix Gla Protein
MPO	Myeloperoxidase
NAG	N-Acetyl-β-d-amino Glycosidase
NC	Nephrocalcin
NGAL	Neutrophil Gelatinase-Associated Lipocalin
OPN	Osteopontin
PCNL	Nephrolithotripsy
PTH	Parathyroid Hormone
RIRS	Retrograde Intrarenal Surgery
SSP	Spontaneous Stone Passage
UPTF-1	Urinary Prothrombin Fragment-1
URS	Ureterorenoscopy
THP	Tamm–Horsfall Protein
TNF	α—Tumor Necrosis Factor Alpha
